# Spherical Minimum Description Length

**DOI:** 10.3390/e20080575

**Published:** 2018-08-03

**Authors:** Trevor Herntier, Koffi Eddy Ihou, Anthony Smith, Anand Rangarajan, Adrian Peter

**Affiliations:** 1 Department of Computer Engineering and Sciences, Florida Institute of Technology, Melbourne, FL 32940, USA; 2 Concordia Institute for Information Systems Engineering, Concordia University, Montreal, QC H3G 1M8, Canada; 3 Department of Computer and Information Science and Engineering, University of Florida, Gainesville, FL 32611, USA

**Keywords:** model selection, MDL, information geometry, von Mises–Fisher distribution, Fisher–Bingham distribution, Fisher information, Laplace approximation, Jeffreys prior

## Abstract

We consider the problem of model selection using the Minimum Description Length (MDL) criterion for distributions with parameters on the hypersphere. Model selection algorithms aim to find a compromise between goodness of fit and model complexity. Variables often considered for complexity penalties involve number of parameters, sample size and shape of the parameter space, with the penalty term often referred to as stochastic complexity. Current model selection criteria either ignore the shape of the parameter space or incorrectly penalize the complexity of the model, largely because typical Laplace approximation techniques yield inaccurate results for curved spaces. We demonstrate how the use of a constrained Laplace approximation on the hypersphere yields a novel complexity measure that more accurately reflects the geometry of these spherical parameters spaces. We refer to this modified model selection criterion as *spherical MDL*. As proof of concept, spherical MDL is used for bin selection in histogram density estimation, performing favorably against other model selection criteria.

## 1. Introduction

The premise of model selection is to objectively choose, from a set of competing models, one that most parsimoniously obtains a good fit to the observed data. The difficulty arises from the fact that goodness of fit and parsimony are inherently conflicting properties. A more philosophical view is sufficiently captured by Ockham’s razor: “Pluralities are never to be put forward without necessity.” The widely established measure of goodness of fit is the likelihood of the observed data. With this issue settled for the most part, research has focused on how to penalize models that overfit the data. Almost all popular model selection criteria differ primarily on the method of this penalizing factor. Simple penalties can depend on only the number of parameters of the model and perhaps the sample size, while more complex criteria take into account the geometric complexity of the parameter manifold. In this work, we revisit the geometry associated with the complexity measure for the Minimum Description Length (MDL) criterion [1,2]. We note that almost all of this previous development is restricted to unconstrained parameter spaces. In this work, we are mainly interested in model selection criteria when parameters are implicitly constrained.

A simple way to accommodate constraints in parametric models is the explicit removal of the constrained set leaving behind a reduced set of unconstrained parameters. Unfortunately, this is difficult to analytically perform when the constraints are nonlinear. In the present work, we mainly focus on unit vector or hypersphere geometry constraints. We show that a constrained MDL-like criteria can be derived in such situations, referring to this new model selection criterion as *spherical MDL*. We also show that there is no requirement to explicitly reduce the set of parameters to a smaller unconstrained set. Instead, we work with the constraints implicitly, extending MDL naturally to such situations. We argue that this opens up MDL to more interesting and constrained parametric models than hitherto seen in the literature. Before introducing spherical MDL and the general methodology behind constrained parametric spaces, we present a simplified version of the current model complexity landscape.

Paramount to every criterion is the value it places on parametric complexity. When making a decision, however, this value is not the greatest concern. It would be natural to think that if models with few parameters are chosen consistently by a a certain criterion, it must be placing a large complexity penalty on models with many parameters. While this may be true, what is actually happening is that, according to this criterion, when models get more complex the *increase* in penalty is larger, making it more undesirable to choose the next most complicated model. In other words, a *K* parameter model may be considered extremely complex, but if the K+1 parameter model isn’t exceedingly more complex, there is little harm in choosing the K+1 parameter model. Every model selection criterion compares this increase in complexity from one model to the next to the improvement in fit and makes its choice accordingly.

Arguably, the three most widely used selection criteria are Akaike’s information criterion (AIC) and its incarnations [3,4,5], Bayesian information criterion (BIC) [6] and Minimum Description Length (MDL). The AIC criterion is given by

(1) $$AIC=-2\log\;f(X;\hat{\theta })+2K$$

and BIC

(2) $$BIC=-2\log\;f\left(X;\hat{\theta }\right)+K\log\left(N\right),$$

where $X={\left\{x_{i}\right\}}_{i=1}^{N}$ is the observed data, *K* is the cardinality of the parameters in the candidate model, *N* is the sample size and $\log\;f(X;\hat{\theta })$ is the log-likelihood of the model evaluated at the maximum likelihood estimate (MLE) $\hat{\theta }$. In the original forms of Equations (1) and (2), the parameters in the set θ specific to the density *f* are assumed to lie in an Euclidean space, i.e., $\theta \in R^{K}$. The candidate model which minimizes the above in each case will be the appropriate model for the data according to the respective criteria. Both criteria use the negative log-likelihood as the measure for goodness of fit and employ similar complexity penalties that reward paucity of parameters. However, BIC includes the sample size in its penalty term and will tend to choose less complex models as more data is collected. Interestingly, in Equation (1), the penalty term can be derived principally from a bias correction between the unknown true model and approximation by the selected model family (and, for more details, see [7]).

The criticisms of the complexity penalties for AIC and BIC are tied to the failure of either to consider how the parameters interact within the model. This shortcoming was addressed in [1,8,9] with the introduction of the Minimum Description Length principle

(3) $$MDL=-\log\;f\left(X;\hat{\theta }\right)+\frac{K}{2}\log\left(\frac{N}{2\pi }\right)+\log\int \sqrt{\det I(\theta )}d\theta ,$$

where I(θ) is the Fisher information matrix. Even though the predecessors to MDL acted as inspirations, Rissanen approached model selection from a unique perspective, that of information theory as opposed to probability theory. Both schools of thought use data to select an appropriate model that can be used to explain the data. However, where probability models aim at searching for the true underlying distribution that generated the data, MDL merely looks at compressing the data. In fact, Rissanen argues [10] that it is entirely inappropriate to look for this “true” distribution since the existence of it is questionable and, as such, the task of trying to estimate it is impracticable. This leaves MDL with the central idea of finding regularities in data and to use these to compress the data such that the data can be described using less symbols. Data is compressed by means of a code and models that offer shorter code lengths are considered to describe the data better. Even though MDL doesn’t concern itself with finding the “true” model, the search for regularities in the data often results in identifying the distribution which generated the data [10].

In this work, and, as mentioned above, we propose a novel MDL-like criterion specifically designed for models with spherical parameter spaces, i.e., $\theta \in S^{K-1}$. We derive the new criterion by revisiting the geometric derivation of MDL—as opposed to its original code-length inspired formulation—and show how when dealing with spherical parametric spaces one can constrain the Laplace approximation to respect this geometry. The geometric derivation of MDL [11] is predicated on carving up parametric manifolds into disjoint regions within which parametric models are indistinguishable. While this approach *prima facie* looks quite different from the standard MDL code length approach, it is shown that the geometric derivation is entirely equivalent to standard MDL. We begin with this geometric approach in the present work since the carving up of parametric manifolds into disjoint regions can be readily extended to constrained parameter spaces. As we show, for the case of spherical MDL, this results in a new complexity term that penalizes based on the normalizing constant of the Fisher–Bingham distribution [12] generalized appropriately to higher dimensions. The MDL criterion, as it is presently formulated, assumes that parameters lie in a Euclidean $R^{K}$ space and are otherwise unconstrained. Asymptotic analyses based on this model are prone to inaccuracies for spherical models. In the remainder of the paper, we detail the theoretical connections with the original MDL criterion and more importantly offer insight into the interpretation of spherical MDL in the context of distinguishable distributions in the model space.

The remainder of this article is organized as follows: Section 2 covers the works most relevant to the present development, including some historical reflection on various model selection criteria. With an ambitious goal of making the article more self-contained, we briefly recap the background on a Bayesian perspective to model selection in Section 3, paying specific attention to geometric motivations behind the use of the Fisher information matrix. Section 4 details the geometric derivation of MDL in $R^{K}$. An approach not as familiar as the original information theoretic formulation, yet enabling the analogous development of MDL on the hypersphere $S^{K-1}$ is detailed in Section 5. By comparing the developments in $R^{K}$ and $S^{K-1}$, one can readily see where the modifications must be made for the constrained parameter space. Next, in Section 6, we consider a practical application of spherical MDL for selecting the bin width of the ubiquitous histogram. Our experimental results validate the utility of spherical MDL in comparison to other state-of-the-art model selection criteria. We conclude in Section 7 with a summary of the developments and future extensions.

## 2. Related Works

Rissanen’s first offering was an early two part code version of MDL. Originally, the MDL criterion was given by

(4) $$MDL=-\log\;f\left(X;\theta \right)+\frac{K}{2}\log\left(\frac{N}{2\pi }\right)$$

and later evolved to become the three part code seen in Equation (3). Similar to AIC and BIC, this two part code fails to penalize for the geometry of the parameter manifold. The third term in Equation (3) penalizes a model for geometric complexity by incorporating the Riemannian volume [13] of the parameter manifold. MDL deviates from AIC [3,4] and BIC [6] in that its objective is not to search for the underlying true model, but to encode regularities in the data. The difficulty with this is that the optimal distribution in the family is required to describe the data properly, but also requires too much information to be optimal. This motivated the idea of identifying a universally represented distribution from a model family, one that compresses every data set almost as well as the best model for every single unique data set. Rissanen coined the term *stochastic complexity* to describe the code length associated with this universal distribution.

In [14], the normalized maximum likelihood (NML) was shown to be the universal distribution of every model family. Specifically, the probability distribution associated with the NML distribution is

(5) $$p\left(X\right)=\frac{f(X|{\hat{\theta }}_{X})}{\int f(Y|{\hat{\theta }}_{Y})dY},$$

where *X* denotes the collected data, *Y* represents any potential data set that could be observed by the experiment and ${\hat{\theta }}_{X}$ denotes their respective maximum likelihood estimate for *X* with a similar notation used for *Y*. The normalizing constant, $\int f(Y|{\hat{\theta }}_{Y})dY$, for the distribution can be thought of as the sum of all maximum likelihood estimates from all possible data sets the experiment could generate. The code length associated with this distribution is found by taking the negative logarithm of Equation (5)

(6) $$SC=-\log\;f\left(X|{\hat{\theta }}_{X}\right)+\log\int f\left(Y|{\hat{\theta }}_{Y}\right)dY$$

and provides us with the mathematical definition of stochastic complexity. Like all other model selection criteria, the first term is a goodness of fit term and the last term is a penalty for complexity, which is sometimes referred to as the parametric complexity and is independent of the data in the sample set. The model that minimizes the stochastic complexity is the one which MDL would choose as optimal.

As elegant as the NML definition of stochastic complexity is, it is not without its flaws. Mainly, the normalizing integral is usually computationally costly to compute making the NML distribution elusive in general and as such it is difficult to compare the stochastic complexity of competing models. In fact, this normalizing integral may not even be finite, a problem which has been named the *infinity problem* [10]. Several solutions have been proposed to fix the infinity problem [15,16], but only in specific cases. Without a satisfactory solution to the problem in general, the NML definition of stochastic complexity is limited in its practical applicability.

Recognizing these issues, an asymptotic formula for the stochastic complexity was derived for larger sample sizes by Balasubramanian in [11]. A brief proof of this formula will be provided in Section 4. The penalty for complexity in this asymptotic formula can be understood in terms of the geometry of the statistical manifold on which the parameters reside. Briefly, instead of trying to compress data using regularities within it, Balasubramanian defines stochastic complexity as the ratio of the volume of an ellipsoid near the MLE to the volume of the entire manifold. An undesirable model would be one in which this ellipsoid is very small when compared to the volume of the entire manifold. We leverage similar geometric arguments when developing spherical MDL in Section 5.

Prior to Rissanen’s MDL, the Minimum Message Length (MML) was introduced in [17]. Rissanen’s MDL is, at its foundation, similar to MML in the sense that both selection criteria aim at finding the model that minimizes the code length that is used to describe the data. However, MDL and MML differ in two important facets [18]. First, MML assumes a prior distribution over the parameters, whereas MDL does not. Intuitively, this prior distribution requires a code length so the code length terms in MDL are inherently shorter. Secondly, the goal of MML is to find the best specific model for the given data. In fact, MML is almost unconcerned as to which model family the selected model belongs. In contrast, MDL searches just for a model class that minimizes the code length needed to explain the data. Further analysis is required to find which specific model within the class best fits the data.

Our model selection criterion is specifically suited for distributions that have parameters residing on a spherical manifold. In [19,20], it was shown that the parameters for histogram density estimation can appropriately be placed on the hypersphere. In [21,22], while showing that MLE theory can be used to estimate the coefficients for wavelet density estimation, it was shown that the coefficients of any square-root density estimator expanded in an orthogonal series resides on a unit hypersphere. In [23], the normalizing constant for the density function for spherical data (not parameters) was studied in detail. Here, Bingham showed that normalizing distributions on the sphere requires a confluent hypergeometric function of matrix argument. Furthermore, in [24], it was suggested that the Laplace approximation employed in the derivation of the asymptotic version of MDL is erroneous when applied on curved manifolds. Here, we show that the spherical MDL integral is instead equal to the normalizing constant of the Fisher–Bingham distribution when the parameters (not data) are constrained to lie on a hypersphere. Even though it can be difficult to calculate, the work in reference [25] offers efficient numerical ways to estimate the value of this normalizing constant.

As anecdotal empirical evidence of our theoretical development of spherical MDL, Section 6 evaluates its use for histogram optimal bin width selection. The authors in [26] detailed the first use of MDL to find the optimal number of bins for histogram estimation. In this case, stochastic complexity for histograms was developed using the notion of code lengths, which is aligned with Rissanen’s original formulation of MDL. Along with the criteria obtained from the code length, two asymptotic versions of the criteria were developed. These three variants of MDL proved to give results that are comparable with other methods of histogram density estimation. The capabilities of the use of NML in MDL has been explored in [27] where the author applies MDL to histogram density estimators with unequal bin widths. Here, histograms vary based on the location and quantity of cut points within the range of the data. Stochastic complexity is found using the normalized maximum likelihood distribution. In [28], the performance of 11 different bin selection criteria were analyzed, among them variants of AIC, BIC and MDL. Here, all the criteria were used to calculate the optimal number of bins for 19 different density shapes and real data. The densities were chosen to analyze the efficacy of each criterion when recognizing varying characteristics of densities, like skewness, kurtosis and multimodality. The performance of each criterion was measured with two different metrics: Peak Identification Loss and the Hellinger risk. Among these results, it was shown that AIC performs relatively poorly when considering either metric, while BIC and MDL were better performers with MDL performing well with both metrics.

## 3. Bayesian Approach to Model Selection

### 3.1. Comparing Models

Suppose we have the parameter space $\Theta^{K}$, such that for all θ∈Θ we have θ: $\theta^{T}\theta =1$. This places all distributions in this space on the (K−1)-dimensional hypersphere. We assume data $X={\left\{x_{i}\right\}}_{i=1}^{N}$ are a sample realization from the density function f(x;θ) where θ∈Θ and the corresponding likelihood function is given by

(7) $$l\left(\theta ;X\right)=\prod_{i=1}^{N}f\left(x_{i};\theta \right).$$

As in [11], we begin with a Bayesian viewpoint of model selection. Taking the simplest case, suppose we have two candidate models *A* and *B* with the goal of choosing one to represent our data. Let $\theta_{A}$ and $\theta_{B}$ be the parameters for each model, most likely of unequal dimensions. For the moment, assume that the parameter spaces are unconstrained. Later, in Section 6, we enforce constraints on them—specifically hypersphere constraints—and will perform model selection in this space.

We wish to examine the posterior of both models and choose the most likely of the candidate models. By Bayes’ rule, the posterior probability of model *A* is

(8) $$\mathrm{Pr}\left(A|X\right)=\frac{\mathrm{Pr}(A)}{\mathrm{Pr}(X)}\int_{S^{K-1}}l\left(\theta ;X\right)\pi \left(\theta \right)d\theta .$$

Here, Pr(A) is the prior probability of model *A*, π(θ) is a prior density over the model parameters and Pr(X) is a prior density function of the data. Candidate model *B* has a similar expression for its posterior. Henceforth, Pr(X) is ignored since it is a common factor. In addition, we take the prior probabilities of each candidate model to be equal and therefore disregarded. The comparison between two posteriors Pr(A|X) and Pr(B|X) therefore devolves into the comparison of two integrals, one with model parameters $\theta_{A}$ and the other with $\theta_{B}$ with the posterior probability being larger for the larger integral. Thus, our goal is the evaluation and maximization of the integral

(9) $$I\left(X\right)=\int_{S^{K-1}}l\left(\theta ;X\right)\pi \left(\theta \right)d\theta$$

over all valid models.

### 3.2. An Inappropriate Prior

Before evaluating Equation (9), we need to define a prior probability in the parameter space. While a uniform prior seems to be a logical choice [29]—following Laplace’s principle of insufficient reason [30]—it is not reparametrization invariant. That is, choosing a uniform distribution as the prior for a specific parametrization does not guarantee that the prior for all parametrizations will be uniform. Let a model be defined by parameter θ with θ∈[0,1] for the sake of convenience. Assume a uniform prior probability density function given by

(10) p(θ)=1,θ∈[0,1].

Now assume a second parametrization of the parameters, ψ along with a monotonic transformation θ→ψ, i.e., ψ=r(θ). Of course, under this new parametrization, we would want the prior distribution to be uniform as well. The prior probability density function over ψ, p(ψ) from Equation (10) is expressed as

(11) $$p\left(\psi \right)=p\left(\theta \right)\left|\frac{\partial r^{-1}\left(\psi \right)}{\partial \psi }\right|\neq 1$$

in general. Clearly, this is undesirable. In fact, p(ψ)=1 is only guaranteed to be true if the transformation, ψ=r(θ) is a translation, which is a very limited reparametrization. That is, an unbiased prior for an arbitrary parametrization fails to give equal weight to the values of the parameters in other parametrizations. A more appropriate prior would be one with a structure that remains the same regardless of the parametrization used. The motivation for a more appropriate prior can be explained using the geometry of hypothesis testing. Below, after a brief discussion on parameter space geometries and their Fisher information, we will revisit this issue of developing a reparameterization invariant prior and its connection to the MDL criterion.

### 3.3. Geometry of Probabilistic Models

Applying geometrical constructs to statistical models is not a new idea. Rao [31] and Jeffreys [32] pioneered the idea of a measure of the distance between two distributions on a parameter manifold. The usefulness of differential geometry in exploring statistical inference is discussed in even greater detail in [33,34]. Here, geometry is tasked with the challenge of finding a metric to measure distances on a statistical manifold. Distributions that are similar to one another reside closer together on the parametric manifold, as measured by the chosen metric. As such, deciding on the appropriate metric opens up geometrical representations for statistical tests. Even though many metrics can be defined, the Fisher information matrix is a natural metric on a parametric manifold due to its invariance property [35].

The manifold associated with a family of models is populated with many distributions. Let a sample set $X={\left\{x_{i}\right\}}_{i=1}^{N}$ be drawn from one of the distributions. A logical statistical question would be, if someone were just given the data, what the probability is with which they would choose the distribution on the manifold which produced the data. The problem of model selection is to pick the best model given a finite sample. Where one distribution can be mistaken for another, we consider the two distributions to be indistinguishable. Distinguishable distributions then can be defined as two distributions that are sufficiently far enough—as measured by the chosen metric—that the probability of mistaking one distribution for another is reasonably small.

Given two probability distributions *f* and *g* defined on the same manifold, relative entropies between *f* and *g* can be defined as [36]:

(12) $$D\left(f∥g\right)=\int f\left(x\right)\ln\left(\frac{f(x)}{g(x)}\right)dx.$$

The parameter vectors associated with each distribution are $\theta_{f}$ and $\theta_{g}$ (i.e., $f\left(x\right)=p\left(x;\theta_{f}\right)$ and $g\left(x\right)=p\left(x;\theta_{g}\right)$. Employing Stein’s lemma [11] in Equation (12) results in

(13) $$D\left(f∥g\right)\approx \frac{1}{2}\Delta \theta^{T}I\left(\theta \right)\Delta \theta ,$$

where $\Delta \theta =\theta_{f}-\theta_{g}$ and I(θ) is the Fisher information matrix (with details below). This strongly suggests that the Fisher information matrix acts as the natural metric on the parameter manifold.

The above discussion shifts attention from unique sets of parameters to counting the number of distinguishable distributions. For an in-depth discussion of distinguishable distributions, please see [11]. For completeness, we include a brief discussion as follows. While it is true that every single distribution is indexed by a unique parameter vector, there is a region around any individual distribution such that distributions in that region are statistically indistinguishable from one another. That is, there is a reasonable probability of mistaking one of the distributions for a neighboring distribution. The size of this elliptical region depends on the natural metric of the manifold, which is the Fisher information, as well as the sample size, since distributions can be more consistently differentiated with a larger sample size.

### 3.4. Fisher Information

The Fisher information matrix is a measure of how much information about the parameter of interest is available from the data collected. Traditionally, the Fisher information matrix is given by

(14) $$I_{i,j}\left(\theta \right)=\int f\left(x;\theta \right)\frac{\partial }{\partial \theta_{i}}\log f\left(x;\theta \right)\frac{\partial }{\partial \theta_{j}}\log f\left(x;\theta \right)dx,$$

where the index (i,j) represents the appropriate parameter pair of the multivariate parameter vector θ. In this form, the Fisher information matrix is the expectation of the variance of the score vector for the multi-parameter distribution f(x;θ).

There are two alternate forms of the Fisher information, provided certain regularity conditions are satisfied. Firstly, we can compute the Fisher information matrix from the expectation of the Hessian of the log likelihood. Specifically,

(15) $$I_{i,j}\left(\theta \right)=-E\left[\frac{\partial^{2}}{\partial \theta_{i}\partial \theta_{j}}\log f\left(x;\theta \right)\right]=-E\left[H\right],$$

where *H* is the Hessian matrix of the log-likelihood.

Alternatively, the Fisher information can be calculated from the variance of the score function

(16) $$I\left(\theta \right)=\mathrm{Var}(S_{f}\left(x;\theta \right)),$$

where

(17) $$S_{f}\left(x;\theta \right)=\nabla \log f\left(x;\theta \right).$$

The Fisher information matrix can be used to define the Cramer–Rao lower bound of unbiased maximum likelihood estimators, determining the optimal sample size in a statistical experiment. Here, we use it for two closely related ideas. The Fisher information is the foundation for developing Jeffreys prior, a non-informative prior that is reparametrization invariant, which solves the issues raised in Section 3.2. In addition, it provides a natural Riemannian metric for a statistical manifold, which will allow us to find volumes of entire closed manifolds as well as the local volume of distinguishability around a single value of the parameter. These volumes will help to interpret the complexity parameter in the spherical MDL criterion proposed in this paper.

### 3.5. An Appropriate Prior

Armed with this geometric definition of the Fisher information, an appropriate non-informative prior can be chosen again starting with two *K*-dimensional parametrizations θ and ψ. Furthermore, we assume there is a transformation ψ=r(θ) with both *r* and its inverse $r^{-1}$ being differentiable, i.e., *r* is a diffeomorphic map. Consider a density function f(x;θ) with score vector

$$S_{f}\left(x;\theta \right)={\left[\begin{array}{ccc} \frac{\partial }{\partial \theta_{1}}\log f\left(x;\theta \right), & \cdots , & \frac{\partial }{\partial \theta_{K}}\log f\left(x;\theta \right) \end{array}\right]}^{T}.$$

Define the Jacobian transformation matrix for both *r* and *r*^−1^ such that

(18) $$J_{r}\left(\theta \right)J_{r^{-1}}\left(\psi \right)=I_{K},$$

where $I_{K}$ is the K×K identity matrix.

The non-informative prior, which is tantamount to Jeffreys prior [32] is $\pi \left(\theta \right)\propto \sqrt{\det I(\theta )}$. To show that Jeffreys prior is invariant to reparametrization, we consider the transformation proposed above. This reparametrization yields a new density function

(19) $$g\left(x;\psi \right)=f\left(x;r^{-1}\left(\psi \right)\right)\left|\det(J_{r^{-1}}\left(\psi \right))\right|$$

and a new score function

(20) $$S_{g}\left(x;\psi \right)={\left(J_{r^{-1}}\left(\psi \right)\right)}^{T}S_{f}\left(x;r^{-1}\left(\psi \right)\right).$$

The new Fisher information, $\tilde{I}\left(\psi \right)$, for the distribution with respect to the new parametrization is

(21) $$\begin{array}{cc} \tilde{I}\left(\psi \right) & =\mathrm{cov}(S_{g}\left(x;\psi \right))\\ & =\mathrm{cov}\left(J_{r^{-1}}{\left(\psi \right)}^{T}S_{f}\left(x;r^{-1}\left(\psi \right)\right)\right)\\ & ={\left(J_{r^{-1}}\left(\psi \right)\right)}^{T}I\left(r^{-1}\left(\psi \right)\right)J_{r^{-1}}\left(\psi \right). \end{array}$$

The Jeffreys prior for the ψ parametrization is

(22) $$\begin{array}{cc} \tilde{\pi }\left(\psi \right) & \propto \sqrt{\det(\tilde{I}\left(\psi \right))}\\ & =\sqrt{\det\left[{\left(J_{r^{-1}}\left(\psi \right)\right)}^{T}I\left(r^{-1}\left(\psi \right)\right)J_{r^{-1}}\left(\psi \right)\right]}\\ & =\det\left(J_{r^{-1}}\left(\psi \right)\right)\det\sqrt{I_{\theta }\left(r^{-1}\left(\psi \right)\right)}\\ & =\det\left(J_{r^{-1}}\left(\psi \right)\right)\pi \left(r^{-1}\left(\psi \right)\right). \end{array}$$

Since the infinitesimals dθ and dψ also transform using the same Jacobian with $d\psi =\det(J_{r}\left(\theta \right))d\theta$, we get

(23) $$\tilde{\pi }\left(\psi \right)d\psi =\pi \left(\theta \right)d\theta .$$

Therefore, the Jeffreys prior remains unchanged under the reparametrization ψ=r(θ) and the value of Equation (9) is indifferent to different representations of the parameter. With this, we can see that the Fisher information directs us to an appropriate prior to use in the evaluation of Equation (9). Specifically, Jeffreys prior is

(24) $$\pi \left(\theta \right)=\frac{\sqrt{\det(I(\theta ))}}{\int \sqrt{\det(I(\theta ))}d\theta },$$

where $\int \sqrt{\det(I(\theta ))}d\theta$ is necessary in order to normalize the prior. In fact, this normalizing constant can sometimes be the largest shortcoming of Jeffreys prior because, in some cases, the integral may not converge, making the prior improper. If the interval diverges, it is possible to place artificial bounds on the limits of integration in order to make the integral converge. However, this won’t be an issue in spherical MDL since, in many applications, the integral will be in closed form and convergent. Selecting a non-informative prior to evaluate Equation (9) is mathematically preferred, making Jeffreys prior the most suitable choice. However, differential geometry gives an interesting interpretation of the Jeffreys prior which will help explain the complexity penalty in spherical MDL. In Riemannian geometry, Equation (13) yields the squared distance element between two nearby points on a parameter manifold, implying again that the Fisher information is a natural metric for the manifold. This metric tensor can be used to calculate volumes of the manifold. Firstly,

(25) $$V_{M}=\int \sqrt{\det\left(I(\theta )\right)}d\theta$$

measures the Riemannian volume of an entire manifold. This integral is evaluated across all possible values of the parameter and, as such, only depends on the model family. As mentioned, this integral is known in closed form for the hypersphere.

Secondly, we are interested in partitioning the volume of the entire manifold into smaller local volumes encompassing indistinguishable distributions. The number of distributions in each volume need not be the same, but every volume is given an equal prior probability. Essentially then, Jeffreys prior provides a uniform prior with regard to these volumes and not individual parameters. With this, the numerator of Equation (24) represents these volumes on the parameter space. More specifically,

(26) $$V_{d}=\sqrt{\det\left(I(\theta )\right)}d\theta$$

can be thought to represent the *infinitesimal* Riemannian volume local to each distinguishable distribution. The complexity parameter for spherical MDL will be interpreted geometrically with these volumes. The reader is pointed to [33] for a more detailed discussion on the appropriateness of Jeffreys prior for spherical distributions.

## 4. Asymptotic MDL in $R^{K}$

In [11], the author develops an alternative derivation of MDL. Instead of attempting to find shortest code lengths, stochastic complexity is approached from a geometric perspective, which is more aligned with our development of spherical MDL. The author begins with a Bayesian approach to model selection and evaluates Equation (9). Again, given a set of data $X={\left\{x_{i}\right\}}_{i=1}^{N}$, the likelihood of any given model with density f(x;θ) and a *K*-dimensional parameter vector is given in Equation (7) and its average negative log-likelihood is given by

(27) $$L\left(\theta \right)=-\frac{1}{N}\log\left(l\left(\theta ;X\right)\right).$$

Our goal is still to evaluate Equation (9):

(28) $$I\left(X\right)=\int \exp\left\{-NL(\theta )\right\}\pi \left(\theta \right)d\theta .$$

To evaluate the integral in Equation (28), we employ standard Laplace approximation techniques [37]. In order to do so, we first expand the integrand around the maximum likelihood estimate of the parameters $\hat{\theta }$ using a Taylor series approximation. The first order term of the expansion of the likelihood *vanishes* at the MLE, resulting in the integral being modified to

(29) $$I\left(X\right)\approx \exp\left\{-NL(\hat{\theta })\right\}\pi \left(\hat{\theta }\right)\int \exp\left\{-\frac{N}{2}{\left(\theta -\hat{\theta }\right)}^{T}H_{I}\left(\theta -\hat{\theta }\right)\right\}d\theta ,$$

where $H_{I}$ is the Hessian of the *unconstrained* negative log likelihood. (We later distinguish this Hessian from that of the constrained log-likelihood.) Recognizing that the quadratic integral will result in a Gaussian integral (for unconstrained parameters), the final evaluation yields an expression for what Balasubramanian called the razor of the model,

(30) $$RZR=\exp\left\{-NL(\hat{\theta })\right\}\pi \left(\hat{\theta }\right){\left(\frac{{\left(\frac{2\pi }{N}\right)}^{K}}{\det(H_{I})}\right)}^{\frac{1}{2}},$$

where *K* is the cardinality of the parameter set. Please note that the standard Laplace approximation has assumed that our parameter space is $R^{K}$. With the aforementioned substitution, and evaluation of the prior from Equation (24) at the maximum likelihood estimate, the final form of MDL is found by taking the negative log of the razor and is given by

(31) $$\begin{array}{c} MDL=-\log\left(RZR\right)=-\log f\left(X;\hat{\theta }\right)+\frac{K}{2}\log\left(\frac{N}{2\pi }\right)+\log\int \sqrt{\det\left(I(\theta )\right)}d\theta . \end{array}$$

The first term in Equation (31) addresses how well the model fits the data. The second and third terms concern the complexity of the model, which has three facets: the number of dimensions in the model, *K*, the form of the model as given by I(θ) and the domain of the parameter set as implied by the limits of integration on the third term.

During the development of Equation (31), the standard Laplace approximation was employed. The Laplace approximation is widely used to evaluate integrals with a unique global maximum over $R^{K}$. However, the authors in [24] suggested that this approximation needs modification in order to be used on curved spaces. Thus, if Equation (31) is to be used on parameters that lie on a hypersphere, the penalty for overfitting will not be accurate, unless the tails of the integrand are ignored. This is the basis of spherical MDL—an extension of the razor approach to MDL to hyperspherical parameter spaces.

To summarize, spherical MDL addresses certain issues that arise in standard MDL. First, the Fisher information integral must exist and be finite. Since this integral represents the Riemannian volume of the model space, and the volumes of unit hyperspheres are available in closed form, this is usually not an (algebraic) concern for spherical MDL. In addition, if the value of the maximum likelihood estimate resides close to the edge of the parameter space, it becomes difficult to find the volume of the parameter space in the immediate vicinity of the MLE. Of course, if the MLE lies on a symmetric space like a hypersphere, then every parameter lies sufficiently in the interior of the model space, so this is not an issue either. Finally, spherical MDL does not ignore parameter constraints (such as restriction to a hypersphere) thereby resulting in a more accurate but still efficiently computable model complexity.

## 5. Spherical MDL

### 5.1. Derivation of the Spherical MDL Criterion

Geometrically, the concept of penalizing a model for complexity can be interpreted as comparing the volume of the manifold in the vicinity of the model corresponding to the MLE to the volume of the entire parameter manifold. If the candidate model occupies very little space on a manifold, it is considered undesirable. This line of development led to the need to evaluate the integral in Equation (9) while constraining it to a (K−1)-dimensional hypersphere.

First, we use standard constrained optimization to enforce the unit length of the coordinate vectors for the parameters. Let

(32) $$M\left(\theta ,\lambda \right)=L\left(\theta \right)+\frac{\lambda }{N}\left(\theta^{T}\theta -1\right)$$

be the Lagrangian corresponding to the constrained optimization problem. Next, the Lagrange parameter λ is set during the process of obtaining the optimal maximum likelihood estimate $\hat{\theta }$. The bulk of the development below attempts to convince the reader that we can rewrite Equation (9) as

(33) $$I\left(X\right)=\int_{S^{K-1}}\exp\left\{-NM(\theta ,\hat{\lambda })\right\}\pi \left(\theta \right)d\theta$$

with the domain of the integral restricted to coordinate vectors on the unit hypersphere.

To evaluate Equation (33), we employ the Laplace approximation methodology but now with the hypersphere constraint enforced. At the outset, this involves finding $\hat{\theta }$, the maximum likelihood estimate of the parameter vector θ at which *M* is minimized. At this minimum value, *M* is stationary, i.e., $\nabla_{\theta }M=0$. We then expand $M(\theta ,\hat{\lambda })$ around this minimum (with the Lagrange parameter set to a fixed value $\hat{\lambda }$). The resulting expansion is

(34) $$M\left(\theta \right)=M\left(\hat{\theta }\right)+\frac{1}{2}{\left(\theta -\hat{\theta }\right)}^{T}H\left(\theta -\hat{\theta }\right)+O(∥\theta -\hat{\theta }{∥}_{2}^{3}),$$

where *H* is the Hessian of *M* (with the Lagrange parameter λ set to its optimum value $\hat{\lambda }$). We now show that this is a principled approach.

If the maximum likelihood problem had been unconstrained, we could have set θ to its MLE value $\hat{\theta }$, expanded the objective function around $\hat{\theta }$ and then employed Laplace’s approximation to obtain the value of the integral in Equation (9). Since the ML parameters θ are constrained to a hypersphere, this route is closed to us. However, we show below that we can begin with a set of independent coordinates (defining a hypersphere) and then prove that the second order Taylor series expansion in Equation (34) is entirely *equivalent* to the corresponding expansion using independent coordinates. That is, we begin with independent coordinates $\theta_{R}$ and a dependent coordinate $\theta_{K}$ and relate the quadratic form emanating from the Taylor series expansion using a carefully constructed “Hessian” of *M* to a corresponding quadratic form driven by the independent Hessian. The derivation closely follows the more general derivation in [38].

When we use independent coordinates to describe a (K−1)-dimensional hypersphere, we get

(35) $$O\left(\theta_{R}\right)\equiv L\left(\theta_{R},\theta_{K}\left(\theta_{R}\right)\right),$$

where the *K*th parameter $\theta_{K}$ has been explicitly written out as a function of the remaining parameters $\theta_{R}\equiv \left\{\theta_{1},\;\theta_{2},\;\ldots ,\;\theta_{K-1}\right\}$. The new objective function $O(\theta_{R})$ corresponds to substituting $\theta_{K}\left(\theta_{R}\right)$ into the negative log-likelihood objective function $L(\theta_{R},\;\theta_{K})$. The partial derivatives of $O(\theta_{R})$ can then be related to the corresponding ones from $L(\theta_{R},\;\theta_{K}\left(\theta_{R}\right))$. Taking partial derivatives, we obtain

(36) $$\frac{\partial O}{\partial \theta_{k}}=\frac{\partial L}{\partial \theta_{k}}+\frac{\partial \theta_{K}}{\partial \theta_{k}}\frac{\partial L}{\partial \theta_{K}},$$

where the explicit dependence of $\theta_{K}$ on $\theta_{k}$ has been included. The second partials are tedious but straightforward to evaluate:

(37) $$\frac{\partial^{2}O}{\partial \theta_{k}\partial \theta_{l}}=\frac{\partial^{2}L}{\partial \theta_{k}\partial \theta_{l}}+\frac{\partial \theta_{K}}{\partial \theta_{l}}\frac{\partial^{2}L}{\partial \theta_{k}\partial \theta_{K}}+\frac{\partial \theta_{K}}{\partial \theta_{k}}\frac{\partial^{2}L}{\partial \theta_{l}\partial \theta_{K}}+\frac{\partial \theta_{K}}{\partial \theta_{k}}\frac{\partial \theta_{K}}{\partial \theta_{l}}\frac{\partial^{2}L}{\partial \theta_{K}^{2}}+\frac{\partial^{2}\theta_{K}}{\partial \theta_{k}\partial \theta_{l}}\frac{\partial L}{\partial \theta_{K}}.$$

The quadratic form corresponding to the independent coordinates $\theta_{R}$ can in turn (after some simplification) be written as

(38) $$\begin{array}{cc} \sum_{kl}u_{k}u_{l}\frac{\partial^{2}O}{\partial \theta_{k}\partial \theta_{l}} & =\sum_{kl}u_{k}u_{l}\frac{\partial^{2}L}{\partial \theta_{k}\partial \theta_{l}}+2\left(\sum_{k=1}^{K-1}u_{k}\frac{\partial \theta_{K}}{\partial \theta_{k}}\right)\left(\sum_{l=1}^{K-1}u_{l}\frac{\partial^{2}L}{\partial \theta_{l}\partial \theta_{K}}\right)\\ & +{\left(\sum_{k=1}^{K-1}u_{k}\frac{\partial \theta_{K}}{\partial \theta_{k}}\right)}^{2}\frac{\partial^{2}L}{\partial \theta_{K}^{2}}+\frac{\partial L}{\partial \theta_{K}}\sum_{kl}u_{k}u_{l}\frac{\partial^{2}\theta_{K}}{\partial \theta_{k}\partial \theta_{l}}. \end{array}$$

Here, $u={\left[u_{1},\;u_{2},\;\ldots ,\;u_{\left(K-1\right)}\right]}^{T}$ and the double summation indices in $\sum_{kl}$ each range from 1 to (K−1). So far, we have made no contact with our constrained optimization problem. From the Lagrangian

(39) $$M\left(\theta ,\hat{\lambda }\right)=L\left(\theta \right)+\frac{\hat{\lambda }}{N}\left(\sum_{k=1}^{K}\theta_{k}^{2}-1\right),$$

where $\hat{\lambda }$ is the optimal value of the Lagrange parameter, we see that the MLE of θ satisfies the relation

(40) $$\frac{\partial M}{\partial \theta_{k}}=\frac{\partial L}{\partial \theta_{k}}+2\theta_{k}\frac{\hat{\lambda }}{N}=0,$$

with the optimal value of the Lagrange parameter

(41) $$\hat{\lambda }=-\frac{N}{2}\sum_{k=1}^{K}{\hat{\theta }}_{k}\frac{\partial L}{\partial \theta_{k}}{|}_{\theta =\hat{\theta }}$$

obtained by multiplying Equation (40) by $\theta_{k}$, summing over all k∈{1,…,K} and enforcing the constraint $\theta^{T}\theta =1$. Furthermore, Equation (40) gives us a relation connecting $\frac{\partial L}{\partial \theta_{K}}$ and $\hat{\lambda }$. We can also obtain a relation connecting $\frac{\partial \theta_{K}}{\partial \theta_{k}}$ and $\left(\theta_{R},\theta_{K}\right)$ by differentiating the constraint equation $\sum_{k=1}^{K-1}\theta_{k}^{2}+\theta_{K}^{2}=1$ once to get

(42) $$2\theta_{k}+2\theta_{K}\frac{\partial \theta_{K}}{\partial \theta_{k}}=0.$$

This relation holds for all θ on the hypersphere unlike Equation (40) which is valid only at the MLE. Taking second derivatives, we obtain

(43) $$2\delta_{kl}+2\frac{\partial \theta_{K}}{\partial \theta_{k}}\frac{\partial \theta_{K}}{\partial \theta_{l}}+2\theta_{K}\frac{\partial^{2}\theta_{K}}{\partial \theta_{k}\partial \theta_{l}}=0.$$

We now have all the ingredients necessary to evaluate Equation (38) for the constrained problem. Substituting Equations (40), (42) and (43) into Equation (38), we get

(44) $$\begin{array}{cc} \sum_{kl}u_{k}u_{l}\frac{\partial^{2}O}{\partial \theta_{k}\partial \theta_{l}} & =\sum_{kl}u_{k}u_{l}\frac{\partial^{2}L}{\partial \theta_{k}\partial \theta_{l}}-\frac{2}{\theta_{K}}\left(\sum_{k=1}^{K-1}u_{k}\theta_{k}\right)\left(\sum_{l=1}^{K-1}u_{l}\frac{\partial^{2}L}{\partial \theta_{l}\partial \theta_{K}}\right)\\ & +\frac{1}{\theta_{K}^{2}}{\left(\sum_{k=1}^{K-1}u_{k}\theta_{k}\right)}^{2}\frac{\partial^{2}L}{\partial \theta_{K}^{2}}+\frac{2\hat{\lambda }}{N}\sum_{kl}u_{k}u_{l}\left(\delta_{kl}+\frac{\theta_{k}\theta_{l}}{\theta_{K}^{2}}\right). \end{array}$$

This can be reorganized with a view toward our goal of obtaining a quadratic form corresponding to a “Hessian” derived from the Lagrangian in Equation (39). We get

(45) $$\begin{array}{cc} \sum_{kl}u_{k}u_{l}\frac{\partial^{2}O}{\partial \theta_{k}\partial \theta_{l}} & =\sum_{kl}u_{k}u_{l}\left(\frac{\partial^{2}L}{\partial \theta_{k}\partial \theta_{l}}+\frac{2\hat{\lambda }}{N}\delta_{kl}\right)-\frac{2}{\theta_{K}}\left(\sum_{k=1}^{K-1}u_{k}\theta_{k}\right)\left(\sum_{l=1}^{K-1}u_{l}\frac{\partial^{2}L}{\partial \theta_{l}\partial \theta_{K}}\right)\\ & +\frac{1}{\theta_{K}^{2}}{\left(\sum_{k=1}^{K-1}u_{k}\theta_{k}\right)}^{2}\left(\frac{\partial^{2}L}{\partial \theta_{K}^{2}}+\frac{2\hat{\lambda }}{N}\right)\\ & =\sum_{kl}u_{k}u_{l}\frac{\partial^{2}M}{\partial \theta_{k}\partial \theta_{l}}-\frac{2}{\theta_{K}}\left(\sum_{k=1}^{K-1}u_{k}\theta_{k}\right)\left(\sum_{l=1}^{K-1}u_{l}\frac{\partial^{2}M}{\partial \theta_{l}\partial \theta_{K}}\right)+\frac{1}{\theta_{K}^{2}}{\left(\sum_{k=1}^{K-1}u_{k}\theta_{k}\right)}^{2}\frac{\partial^{2}M}{\partial \theta_{K}^{2}}, \end{array}$$

where we have taken care to set $\hat{\lambda }$ to its optimum MLE value (while not treating it as a function of θ). Consequently, the second partials of *Mdo not* include the dependence of $\hat{\lambda }$ on $\hat{\theta }$. To further simplify this expression, we now define $v\equiv {\left[u_{1},\;u_{2},\;\ldots ,\;u_{\left(K-1\right)},-\frac{1}{\theta_{K}}\sum_{k=1}^{K-1}u_{k}\theta_{k}\right]}^{T}$. Note that *v* satisfies the constraint $\sum_{k=1}^{K}v_{k}\theta_{k}=0$ implying that *v* is orthogonal to θ. This will be important later on in the specification of the constrained quadratic form. Using the definition of the Lagrangian in Equation (39), we get

(46) $$\sum_{kl}u_{k}u_{l}\frac{\partial^{2}O}{\partial \theta_{k}\partial \theta_{l}}{|}_{\theta =\hat{\theta }}=\sum_{k=1}^{K}\sum_{l=1}^{K}v_{k}v_{l}\frac{\partial^{2}M}{\partial \theta_{k}\partial \theta_{l}}{|}_{\theta =\hat{\theta }},$$

which implies the equality of the independent and constrained quadratic forms. Note that the constraint $\sum_{k=1}^{K}\theta_{k}^{2}=1$ implies that

(47) $$\sum_{k=1}^{K}\theta_{k}d\theta_{k}=0,$$

where $d\theta_{k}$ is an infinitesimal quantity. Assuming this remains valid for a small (but not infinitesimal) vector Δθ (up to second order correction factors), this in turn implies that the increment vector ${\left[\Delta \theta_{1},\;\Delta \theta_{2},\;\ldots ,\;\Delta \theta_{K}\right]}^{T}$ is orthogonal to the gradient of the constraints, equal to ${\left[2\theta_{1},\;2\theta_{2},\;\ldots ,\;2\theta_{K}\right]}^{T}$. Therefore, the quadratic form obtained from the Lagrangian *M* is only valid in the subspace spanned by increment vectors $\left\{v|\sum_{k=1}^{K}v_{k}\theta_{k}=0\right\}$. This further implies that this quadratic form is equivalent to the independent quadratic form in Equation (38) provided the increments are confined to the correct subspace.

Given the above analysis, the second order Taylor series expansion of *M* around the MLE estimate $\hat{\theta }$ in Equation (34), where the (k,l) element of the Hessian is

(48) $$H_{kl}=\frac{\partial^{2}M}{\partial \theta_{k}\partial \theta_{l}}{|}_{\theta =\hat{\theta }}=\frac{\partial^{2}L}{\partial \theta_{k}\partial \theta_{l}}{|}_{\theta =\hat{\theta }}+\frac{2\hat{\lambda }}{N}\delta_{kl},$$

emerges as the quantity most closely connected to the expansion of the independent objective *O* using coordinates $\theta_{R}$. When the increments $\theta -\hat{\theta }$ are confined to the subspace orthogonal to the gradient vector ${\left[2{\hat{\theta }}_{1},\;2{\hat{\theta }}_{2},\;\ldots ,\;2{\hat{\theta }}_{K}\right]}^{T}$, i.e.,

(49) $$\sum_{k=1}^{K}2\left(\theta_{k}-{\hat{\theta }}_{k}\right){\hat{\theta }}_{k}=0,$$

then the quadratic form ${\left(\theta -\hat{\theta }\right)}^{T}H\left(\theta -\hat{\theta }\right)$ is equivalent to the independent one as shown above in Equation (46). In the subsequent calculations, we set $\hat{\theta }$ to the constrained maximum likelihood solution (wherein $\hat{\theta }$ is constrained to lie on the surface of a unit hypersphere) and allow θ to vary over just the surface of the same unit hypersphere. For values of θ close to $\hat{\theta }$, $\theta -\hat{\theta }$ will approximately satisfy Equation (49), thereby validating our choice of “Hessian” for the hyperspherically constrained Laplace approximation.

A question may arise at this juncture as to why we could not have directly worked with the independent coordinates in the first place. Insofar as parameter constraints remain implicit (and hypersphere constraints fall into this category), it is much easier to work with constrained and implicit parameterizations than explicit ones (since the latter are typically harder to come by). Provided the manifold integrals can be carried out without defaulting to Gaussian integrals—and we make this case throughout the present work—implicit parameterizations should be preferred, especially given the correspondence worked out above between the constrained and independent quadratic forms.

With the Hessian defined in this manner (and related to the Lagrangian *M*), an asymptotic solution to Equation (33) can be found. Since Equation (32) represents the Lagrangian as a Taylor expansion around the MLE, it will be useful to redefine the entire integrand as a Taylor expansion. As such, the prior, $\overset{´}{\pi (\theta )}$, needs to be expanded as well. Expanding the prior around the MLE, we get

(50) $$\pi \left(\theta \right)=\pi \left(\hat{\theta }\right)+{\left(\theta -\hat{\theta }\right)}^{T}\nabla \pi \left(\hat{\theta }\right)+O(∥\theta -\hat{\theta }{∥}_{2}^{2}).$$

We now rewrite Equation (33) as a product of Equations (34) and (50) to get

(51) $$\begin{array}{cc} I(X) & =\int_{S^{K-1}}\exp\left\{-NM(\theta ,\hat{\lambda }\right\}\pi \left(\theta \right)d\theta \\ & \approx \int_{S^{K-1}}\exp\left[-NM\left(\hat{\theta }\right)-\frac{N}{2}{\left(\theta -\hat{\theta }\right)}^{T}H\left(\theta -\hat{\theta }\right)\right]\left[\pi \left(\hat{\theta }\right)+{\left(\theta -\hat{\theta }\right)}^{T}\nabla \pi \left(\hat{\theta }\right)+\cdots \right]d\theta \\ & \approx \exp\left\{-N(M\left(\hat{\theta }\right)\right\}\pi \left(\hat{\theta }\right)\int_{S^{K-1}}\exp\left\{-\frac{N}{2}{\left(\theta -\hat{\theta }\right)}^{T}H\left(\theta -\hat{\theta }\right)\right\}d\theta . \end{array}$$

Here, we have used the fact that $\theta \to \hat{\theta }$ as N→∞ on the order of $N^{-\frac{1}{2}}$ [39] which makes

(52) $$\int_{S^{K-1}}{\left(\theta -\hat{\theta }\right)}^{T}\nabla \pi \left(\hat{\theta }\right)d\theta =0.$$

The evaluation of the integral of the quadratic term in Equation (51), when constrained to the (K−1)-dimensional hypersphere, is where Rissanen’s MDL *inaccurately* penalizes the stochastic complexity of spherical parameter spaces. Instead of resulting in a Gaussian integral, there is no closed form solution in general. However, for distributions whose individual parameters contribute equally to the Fisher information matrix, as is the case in the histogram, we can efficiently evaluate this integral. This assertion will be expanded upon in Section 6.

We continue solving Equation (51), and using the Jeffreys prior as the appropriate prior, we get

(53) $$I\left(X\right)=\exp\left\{-NM(\hat{\theta })\right\}\int_{S^{K-1}}\exp\left\{-\frac{N}{2}{\left(\theta -\hat{\theta }\right)}^{T}H\left(\theta -\hat{\theta }\right)\right\}d\theta \frac{\sqrt{\det(I\left(\hat{\theta }\right))}}{\int \sqrt{\det(I(\theta ))}d\theta }.$$

As is customary with most model selection criteria, the optimal model according to spherical MDL will be the one which minimizes the −log of Equation (53). Hence,

(54) $$\begin{array}{cc} MDL_{S^{K-1}} & =\;NM\left(\hat{\theta }\right)-\log\sqrt{\det(I\left(\hat{\theta }\right))}+\log\int \sqrt{\det(I(\theta ))}d\theta \\ & -\log\int_{S^{K-1}}\exp\left\{-\frac{N}{2}{\left(\theta -\hat{\theta }\right)}^{T}H\left(\theta -\hat{\theta }\right)\right\}d\theta \\ & =\;N\left[L+\hat{\lambda }\left({\hat{\theta }}^{T}\hat{\theta }-1\right)\right]-\log\sqrt{\det(I\left(\hat{\theta }\right))}+\log\int \sqrt{\det(I(\theta ))}d\theta \\ & -\log\int_{S^{K-1}}\exp\left\{-\frac{N}{2}{\left(\theta -\hat{\theta }\right)}^{T}H\left(\theta -\hat{\theta }\right)\right\}d\theta \\ & =\;-\log l\left(\hat{\theta }\right)-\log\sqrt{\det(I\left(\hat{\theta }\right))}+\log\int \sqrt{\det(I(\theta ))}d\theta \\ & -\log\int_{S^{K-1}}\exp\left\{-\frac{N}{2}{\left(\theta -\hat{\theta }\right)}^{T}H\left(\theta -\hat{\theta }\right)\right\}d\theta . \end{array}$$

The first term in Equation (54) is the log-likelihood and rewards a model for goodness of fit. The last three terms represent the parametric complexity penalty in spherical MDL:

(55) $$\begin{array}{c} C=-\log\sqrt{\det(I\left(\hat{\theta }\right))}+\log\int \sqrt{\det(I(\theta ))}d\theta -\log\int_{S^{K-1}}\exp\left\{-\frac{N}{2}{\left(\theta -\hat{\theta }\right)}^{T}H\left(\theta -\hat{\theta }\right)\right\}d\theta . \end{array}$$

The complexity penalty reflects the proportion of the volume of the total parameter space that lies close to the one model that best describes the data. The second term in Equation (55) is independent of the data and the candidate model, and therefore must reflect only the complexity in the inherent chosen model family. Specifically, this term represents the volume of the parameter manifold which is known in closed form. The first term represents the local volume around the model corresponding to the MLE, as measured by the natural measure of the parameter manifold. The final term is dependent upon the intrinsic properties of the model family, attributes of the data and on the candidate distribution. Essentially, it measures the volume of an ellipsoid around the parameter with respect to a local metric determined by the data. The essence of spherical MDL is within this integral. During the course of the evaluation of this integral, the small ellipse around the MLE is constrained to lie on the surface of the sphere.

Alternatively, the complexity term in Equation (55) can be represented as a ratio of two terms

(56) $$C=-\log\left[\frac{\sqrt{\det(I\left(\hat{\theta }\right))}\int_{S^{K-1}}\exp\left\{-\frac{N}{2}{\left(\theta -\hat{\theta }\right)}^{T}H\left(\theta -\hat{\theta }\right)\right\}d\theta }{\int \sqrt{\det(I(\theta ))}d\theta }\right].$$

Here, the denominator is the volume of the entire parameter manifold. The numerator is the volume of a small ellipsoid on the surface of the sphere around the MLE. If the volume around the MLE is small compared to the volume of the entire manifold, the model is considered complex and this term grows accordingly.

In contrast, the complexity penalty for the asymptotic version of Rissanen’s MDL in $R^{K}$ is

(57) $$C_{R^{K}}=\frac{K}{2}\log\left(\frac{N}{2\pi }\right)+\log\int \sqrt{\det\left(I(\theta )\right)}d\theta .$$

### 5.2. Riemannian Volume of a Hypersphere

The asymptotic version of MDL requires that the entire Riemannian volume of the manifold be finite. That is, $\int \sqrt{\det(I(\theta ))}d\theta$ must converge. In complicated cases, this can be very impractical. If the manifold is unbounded, compromises such as artificially bounding the parameter space are required in order for the integral $\int \sqrt{\det(I(\theta ))}d\theta$ to converge. Approximations using Monte Carlo integration are also utilized [40]. As difficult as this mathematical hurdle can be to overcome, it ends up being a big advantage for spherical MDL. In many cases, spherical MDL concerns itself with hyperspherical manifolds with Riemannian volumes equivalent to the surface area of a (K−1)-dimensional hypersphere, which is known in closed form.

The equation for the volume of a hypersphere is

(58) $$V_{M}=\left\{\begin{array}{cc} K\pi^{K/2}, & K\;\mathrm{even},\\ 2^{K}\pi^{\frac{K-1}{2}}\frac{\left(\frac{K-1}{2}\right)!}{\left(K-1\right)!}, & K\;\mathrm{odd}. \end{array}\right.$$

**Figure 1:**
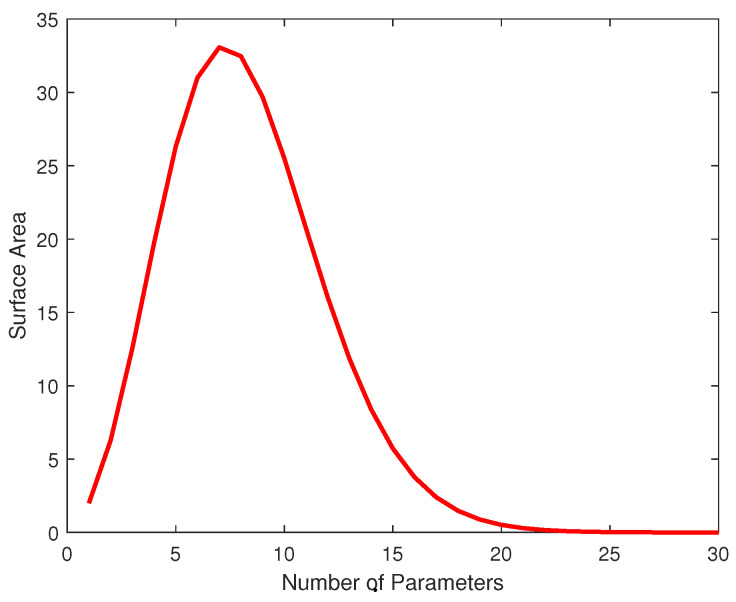
Riemannian Volume of Hypersphere. The surface area of a hyperspherical manifold plotted against the cardinality of the parameters. Interestingly, the surface area grows to a maximum at seven dimensions and then monotonically decreases. Accordingly, a seven-dimensional model family requires a relatively large ellipsoid around the MLE in order to avoid excessive penalties for complexity.

Figure 1 shows the volume of the unit hypersphere (technically the surface area) as a function of dimension. Curiously, this volume reaches a maximum at seven parameters after which the volume rapidly decreases approaching 0. Having the volume of the entire manifold decreasing to 0 can be troublesome since on the manifold, there must be room for a local volume around distinguishable distributions. In fact, in some cases, the local volume around a parameter can exceed the volume of the entire parameter space resulting in misspecified models. However, high dimensional models only make sense when dealing with large sample sizes. In this scenario, the dissimilarity between two neighboring distributions becomes more noticeable. This allows the volume around each detectable distribution to shrink. Thus, as the number of parameters increases, the volume of the entire manifold decreases. However, it only makes sense to use these models with sample sizes that are large enough to force the volume around the MLE to be smaller than the overall manifold’s volume [41]. Since spherical MDL represents an asymptotic approximation of NML, it is more suitable when applied to large sample data. We refer the reader to [42,43] for more details regarding the issue of misspecification for high-dimensional models.

## 6. Case Study: Spherical MDL for Histograms

The regular histogram is one of the most popular nonparametric density estimators. It is the go-to method for data scientists for quickly visualizing the regularities of their data. With relatively few parameters, a histogram can approximately model a variety of density functions without explicit knowledge of any underlying structure. Despite their simplicity, histograms can display very complicated characteristics of density functions like kurtosis and multimodality, which are often tied to the construction method.

Histogram construction always begs the question: what are the appropriate number of bins? While, in most cases, since the ultimate purpose of the histogram is to highlight features in the data, a subjective choice of number of bins would be one that best shows the features you wish to highlight. However, it is possible to use model selection to remove some of the subjectivity from this decision. Here, as a simple proof of concept, we show how spherical MDL can be used to select the optimal number of bins for a fixed-bin-width histogram. We detail the full scope of applying spherical MDL to this model, starting from the log likelihood and then deriving the relevant equations from Section 5 to reach the final criterion given in Equation (54).

Probably because of its prominence, approaches to bin selection for histograms are very popular, with many of the schemes deeply rooted in model selection theory [28,44]. Here, we consider histograms with equal bin width, also known as regular histograms. When doing so, we can use Equation (54) to optimize the number of bins once a simple algebraic transformation produces the required hypersphere geometry. The geometric interpretation of spherical MDL also allows for a satisfying solution to the question of how to penalize empty bins, something ignored in much of the current research or addressed by allowing for unequal bin width.

### 6.1. Theoretical Development

The histogram can be realized by estimating an unknown density function via deploying piecewise constant functions and then using the maximum likelihood estimator, which results in the histogram. The height of each bin is proportional to the number of data points falling in its interval, i.e.,

(59) $$f\left(x\right)=\left\{\begin{array}{cc} c_{i}, & \mathrm{if}\;x\;\mathrm{is}\;\mathrm{in}\;\mathrm{interval}\;i,\\ 0, & \mathrm{otherwise}. \end{array}\right.$$

Given data $X=\{x_{1},\;x_{2},\;\ldots ,\;x_{N}\}$, the likelihood function is given by

(60) $$l\left(c\right)=\prod_{i=1}^{K}c_{i}^{v_{i}},$$

where $v_{i}$ is the number of data points in the *i*-th interval and *K* is the number of bins.This makes the average negative log-likelihood

(61) $$L\left(c\right)=-\frac{1}{N}\sum_{i=1}^{K}v_{i}\log c_{i}.$$

As in [20], we choose to map the parameters of the histogram to the hypersphere. We begin by making the variable substitution $u_{i}^{2}=c_{i}$ after which the average negative log-likelihood becomes (with a mild abuse of notation)

(62) $$L\left(u\right)=-\frac{1}{N}\sum_{i=1}^{K}2v_{i}\log u_{i}.$$

We now restrict the parameters to lie on a (K−1)-dimensional hypersphere by setting

(63) $$\sum_{i=1}^{K}u_{i}^{2}=h^{-1},$$

where *h* is the regular bin width of the histogram. This ensures the volume under the density to be one. To emphasize the dependence of the complexity on the number of parameters *K*, we make the substitution $h=\frac{R}{K}$, where *R* is the range of the data. The constrained average negative log-likelihood is then

(64) $$\begin{array}{cc} M(u,\lambda ) & =L\left(u\right)+\frac{\lambda }{N}\left(\sum_{i=1}^{K}u_{i}^{2}-\frac{K}{R}\right)\\ & =-\frac{1}{N}\left[\sum_{i=1}^{K}2v_{i}\log u_{i}-\lambda \left(\sum_{i=1}^{K}u_{i}^{2}-\frac{K}{R}\right)\right]. \end{array}$$

Minimizing M(u,λ) with respect to *u* yields ${\hat{u}}_{k}=\sqrt{\frac{v_{k}K}{NR}}$ with the optimal value of the Lagrange parameter being $\hat{\lambda }=N\frac{R}{K}.$

We wish to solve Equation (48) for the histogram density log-likelihood function Equation (64). Starting with Equation (62),

(65) $$L\left(u\right)=-\frac{1}{N}\sum_{i=1}^{K}2v_{i}\log u_{i},$$

the resulting gradient is

(66) $$\frac{\partial L}{\partial u_{k}}=-\frac{1}{N}\frac{2v_{k}}{u_{k}}$$

and the Hessian

(67) $$\frac{\partial^{2}L}{\partial u_{k}^{2}}=\frac{1}{N}\frac{2v_{k}}{u_{k}^{2}}$$

with all other mixed partials equal to zero.

Next, we evaluate the Hessian of the average negative log likelihood at the MLE. Using Equation (48),

(68) $$\begin{array}{cc} H_{kk} & =\frac{2v_{k}}{\frac{Kv_{k}}{R}}+2\frac{R}{K}\\ & =4\frac{R}{K}. \end{array}$$

Hence, the Hessian is a diagonal matrix with all positive entries, ensuring that it is positive definite as required by the Taylor expansion in Equation (48). To evaluate the Fisher information, we take the expectation of Equation (68) to get

(69) $$\begin{array}{cc} I_{kk}\left(\theta \right) & =\int H_{kk}f\left(x\right)dx\\ & =H_{kk}\int f\left(x\right)dx\\ & =4\frac{R}{K}, \end{array}$$

which does not depend on the histogram model parameters.

The parameters of the histogram density function do not lie on a unit hypersphere but, rather, they reside on a hypersphere with radius ${\left(\frac{K}{R}\right)}^{\frac{1}{2}}$. Additionally, this volume requires a scale factor of $\sqrt{\det(I\left(\hat{\theta }\right))}={\left(4\frac{R}{K}\right)}^{\frac{K}{2}}$ based on the Fisher information from Equation (69) which defines a metric tensor on our manifold. Considering both of these influences, the volume of our sphere must be adjusted by a factor of ${\left(\frac{K}{R}\right)}^{\frac{K}{2}}{\left(4\frac{R}{K}\right)}^{\frac{K}{2}}=2^{K}$, making the volume of the entire manifold for each family

(70) $$V_{H}=2^{K}V_{M},$$

where $V_{M}$ is the hypersphere volume given in Equation (58). It may seem necessary to restrict the volume of the manifold even further, considering that the parameters of the histogram necessarily reside only in the positive hyperorthant of the hypersphere and the volume in Equation (70) accounts for the entire hypersphere. However, the same logic that would apply to restricting the Riemannian volume would also apply to the integral of the exponential of the quadratic form in Equation (54). With both restrictions having the same opposite effects on spherical MDL, restriction to the positive hyperorthant becomes unnecessary.

According to spherical MDL, the optimal number of bins for a histogram is the one which minimizes

(71) $$\begin{array}{cc} MDL_{sphere} & =-\sum_{i=1}^{K}2v_{i}\log u_{i}-\log\sqrt{\det(I\left(\hat{\theta }\right))}+\log V_{H}\\ & -\log\int_{S^{K-1}}\exp\left\{-\frac{N}{2}{\left(\theta -\hat{\theta }\right)}^{T}H\left(\theta -\hat{\theta }\right)\right\}d\theta \\ & =-\sum_{i=1}^{K}2v_{i}\log u_{i}-\frac{K}{2}\log\left(4\frac{R}{K}\right)+\log V_{H}\\ & -\log\int_{S^{K-1}}\exp\left\{-\frac{N}{2}{\left(\theta -\hat{\theta }\right)}^{T}H\left(\theta -\hat{\theta }\right)\right\}d\theta . \end{array}$$

The third term, which is independent of the data, penalizes solely based on the number of parameters in the model family. If the model family being assessed has *K* parameters, of which *l* are empty, the model family is penalized as a *K* parameter family and *not* as a K−l parameter family. This particular distribution with *l* empty bins simply is one which resides on the *l* axes of the hypersphere.

The final term can be elusive to find in general, but when the Hessian of the log-likelihood consists of identical elements as it does with the histogram, the integral now represents the normalizing constant of the von Mises distribution whose solution is known in closed form. Focusing just on this integral, we first expand the quadratic form, recalling that every diagonal element of the Hessian is 4h and can be amalgamated with $\frac{N}{2}$ to get

(72) $$\begin{array}{cc} Q(\hat{\theta }) & =\int_{S^{K-1}}\exp\left\{-\frac{N}{2}{\left(\theta -\hat{\theta }\right)}^{T}H\left(\theta -\hat{\theta }\right)\right\}d\theta \\ & =\int_{S^{K-1}}\exp\left\{-2Nh{\left(\theta -\hat{\theta }\right)}^{T}\left(\theta -\hat{\theta }\right)\right\}d\theta \\ & =\int_{S^{K-1}}\exp\left\{-2Nh(\theta^{T}\theta -2\theta^{T}\hat{\theta }+{\hat{\theta }}^{T}\hat{\theta })\right\}d\theta . \end{array}$$

Now, in the expanded quadratic, we have two quadratic terms that are subject to our constraint $\theta^{T}\theta =h^{-1}$. We can further simplify the integral to be

(73) $$\begin{array}{cc} Q(\hat{\theta }) & =\int_{S^{K-1}}\exp\left\{-2Nh(h^{-1}-2\theta^{T}\hat{\theta }+h^{-1})\right\}d\theta \\ & =\int_{S^{K-1}}\exp\left\{-4N+4Nh\theta^{T}\hat{\theta })\right\}d\theta \\ & =\exp\left\{-4N\right\}\int_{S^{K-1}}\exp\left\{4Nh\theta^{T}\hat{\theta })\right\}d\theta . \end{array}$$

In order to satisfy the definition of the von Mises distribution, we will need to put this on the unit hypersphere. We do this by making the following substitutions:

(74) $$\frac{x_{i}}{\sqrt[{}]{h}}=\theta_{i},\frac{\hat{x_{i}}}{\sqrt[{}]{h}}=\hat{\theta_{i}}\;\mathrm{and}\;d\theta =\frac{dx}{\sqrt[{}]{h}}.$$

Once again, to more clearly show that complexity increases as the number of parameters increases, we make the substitution $h=\frac{R}{K}$. Equation (73) becomes (after a minor abuse of notation)

(75) $$\begin{array}{cc} Q(\hat{x}) & =\exp\left(-4N\right)\int_{S^{K-1}}\exp\left\{4Nh\frac{x^{T}}{\sqrt[{}]{h}}\frac{{\hat{x}}^{T}}{\sqrt[{}]{h}}\right\}dx\frac{1}{{\sqrt[{}]{h}}^{K}}\\ & ={\left(\frac{K}{R}\right)}^{\frac{K}{2}}\exp\left(-4N\right)\int_{S^{K-1}}\exp\left\{4Nx^{T}\hat{x}\right\}dx. \end{array}$$

The integral in Equation (75) is now in the form of a von Mises distribution. In general, the von Mises distribution is

(76) $$f_{K}\left(x,u,\kappa \right)=\frac{\exp(\kappa u^{T}x)}{C_{K}\left(\kappa \right)},$$

where κ≥0, ∥u∥=1 and *x* is random unit vector. The distribution in Equation (76) must integrate to one, so

(77) $$\int_{S^{K-1}}\exp\left(\kappa u^{T}x\right)dx=C_{K}\left(\kappa \right),$$

where

(78) $$C_{K}\left(\kappa \right)={\left(\frac{2\pi }{\kappa }\right)}^{\frac{K}{2}}\kappa \;I_{\frac{K}{2}-1}\left(\kappa \right)$$

and $I_{\zeta }\left(\kappa \right)$ is the modified Bessel function of order ζ [45]. The right side of Equation (78) will be used to determine the value of the integral in Equation (75).

By comparing the integral in Equation (75) to Equation (77), we can see that κ=4N. With this, Equation (75) then becomes

(79) $$Q\left(\hat{x}\right)={\left(\frac{K}{R}\right)}^{\frac{K}{2}}\exp\left(-4N\right){\left(\frac{\pi }{2N}\right)}^{\frac{K}{2}}4N\;I_{\frac{K}{2}-1}\left(4N\right),$$

which is independent of $\hat{x}$. Substituting this into Equation (71), we obtain that spherical MDL will choose a histogram with the number of bins that minimizes

(80) $$\begin{array}{cc} MDL_{sphere} & =-\sum_{i=1}^{K}2v_{i}\log u_{i}-\frac{K}{2}\log\left(4\frac{R}{K}\right)+\log V_{H}\\ & -\log\int_{S^{K-1}}\exp\left\{-\frac{N}{2}{\left(\theta -\hat{\theta }\right)}^{T}H\left(\theta -\hat{\theta }\right)\right\}d\theta \\ & =-\sum_{i=1}^{K}2v_{i}\log u_{i}-\frac{K}{2}\log\left(4\frac{R}{K}\right)+\log V_{H}\\ & -\log\left[{\left(\frac{K}{R}\right)}^{\frac{K}{2}}\exp\left(-4N\right){\left(\frac{\pi }{2N}\right)}^{\frac{K}{2}}4N\;I_{\frac{K}{2}-1}\left(4N\right)\right], \end{array}$$

where $V_{H}$ is defined in Equation (70). We note in passing that, even though terms that only depend on the sample size will contribute to the complexity of the model, they don’t contribute to the selection process since they are identical to every model. With this, Equation (80) simplifies to

(81) $$\begin{array}{cc} MDL_{sphere} & =-\sum_{i=1}^{K}2v_{i}\log u_{i}+\frac{K}{2}\log\left(\frac{K}{4R}\right)+\log V_{H}\\ & +\frac{K}{2}\log\left(\frac{R}{K}\right)+\frac{K}{2}\log\left(\frac{2N}{\pi }\right)-\log\left(\;I_{\frac{K}{2}-1}\left(4N\right)\right)\\ & =-\sum_{i=1}^{K}2v_{i}\log u_{i}+\log\left(V_{H}\right)+\frac{K}{2}\log\left(\frac{N}{2\pi }\right)-\log\left(I_{\frac{K}{2}-1}\left(4N\right)\right). \end{array}$$

Spherical MDL closely tracks ordinary MDL when it comes to asymptotics. The modified Bessel function in Equation (81) can be considerably simplified as N→∞:

(82) $$I_{\frac{\left(K-1\right)}{2}}\left(4N\right)\approx \frac{\exp\{4N\}}{\sqrt{2\pi }}\left[\frac{1}{{\left(4N\right)}^{\frac{1}{2}}}+\frac{\left(4K-3-K^{2}\right)}{8{\left(4N\right)}^{\frac{3}{2}}}+O\left(\frac{1}{{\left(4N\right)}^{\frac{5}{2}}}\right)\right].$$

Since the leading term in Equation (82) is independent of *K*, we obtain that spherical MDL and ordinary MDL converge to the same complexity (after ignoring terms independent of *K*) as N→∞.

### 6.2. Experimental Results

Every model selection criterion uniquely penalizes parametric complexity. All penalties have mathematical foundations that validate their individual appropriateness. In the case of choosing a model for a distribution whose parameters lie on the hypersphere, as is the case for the histogram, criteria that ignore the geometry of the manifold or improperly apply asymptotic approximations are inherently less appropriate than a criterion that considers these characteristics.

**Figure 2:**
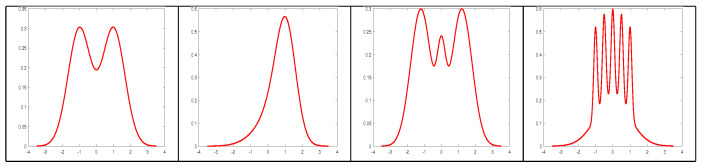
Four different densities selected for varying characteristics. Bimodal (left), skewed (center left), trimodal (center right) and claw (right).

Experiments were conducted generating results of optimal bin counts for histograms of differently shaped distributions. A variety of sampling distributions were created from mixtures of one-dimensional Gaussian distributions as in [46,47]. The densities chosen represent many characteristics of real densities such as multimodality, skewness and spatial variability. The densities estimated were: Bimodal, Skewed Unimodal, Trimodal and Claw as shown in Figure 2. In addition, 2500 trials of sample size 60 were taken from each distribution. The optimal number of bins for AIC, BIC, two part MDL from Equation (4), Balasubramanian’s asymptotic MDL from Equation (31) and spherical MDL was calculated for each trial. The frequency with which the decisions made by AIC, BIC, two part MDL and asymptotic MDL deviated from the decision made by spherical MDL are summarized in Table 1.

The results show that AIC and two part MDL penalize complex models the least with AIC most frequently making incorrect decisions. This is true to the reputation of AIC, at reasonable sample sizes. This is expected considering that the number of parameters alone are used to penalize models, with sample size not considered. BIC always chooses models that are the least complex, showing the importance it places on sample size. Balasubramanian’s asymptotic MDL and spherical MDL always choose models that have less extreme number of bins. When compared to MDL, spherical MDL tended to prefer less complex models, indicating that MDL underpenalizes the complexity of curved parameter spaces. While these results are somewhat anecdotal, they serve to demonstrate the importance of incorporating the histogram’s hypersphere geometry into model selection. Furthermore, in general, we advocate for the modification of model selection criteria to respect their parameter space geometries.

## 7. Conclusions

Model selection criteria seek to parsimoniously balance complexity and goodness of fit. Though many formulations exist, like the well-known AIC, BIC and ordinary MDL, most of them fail to appropriately consider the geometry of the parameter manifold when penalizing models. This always results in underpenalizing the complexity for AIC (for example). Here, we have revisited the MDL criterion from a geometric perspective and derived a new measure for spherical parameter spaces.

Spherical MDL incorporates appropriate asymptotic and geometric arguments to ensure the resulting criterion is intrinsic to the manifold. It was shown through experimental trials that, if regular MDL is used, the complexity penalty is small, resulting in choosing optimal models that are somewhat more complex than spherical MDL. The complexity penalty of the proposed spherical MDL measure employs corrections that take into consideration the shape of the manifold and mitigates the tendency to select unnecessarily complicated models. Though this present effort focused on spherical parameter manifolds, our geometric approach to model selection can be generalized to other curved domains.

## Figures and Tables

**Table 1:** Frequency of deviation of 2500 trials of the choice made by Akaike’s information criterion (AIC), Bayesian information criterion (BIC), two part Minimum Description Length (MDL2) and asymptotic MDL (MDL) from the choice of spherical MDL for a sample size of 60 drawn from different distributions. We found that BIC consistently penalizes complexity the most while AIC and MDL2 are consistently forgiving of complex models. Spherical MDL and ordinary MDL offer a compromise between goodness of fit and complexity, with spherical MDL always choosing a less complex model, showing that ordinary MDL underpenalizes the complexity of curved parameter spaces.

	AIC	BIC	MDL2	MDL
Bimodal	1407	221	1372	4
Skew	1441	200	1349	9
Trimodal	1478	197	1323	3
Claw	1569	257	1471	6
Total	5895	875	5515	22

## References

[1] Rissanen J. (1978). Modeling by shortest data description. Automatica.

[2] Rissanen J. (1983). A universal prior for integers and estimation by minimum description length. Ann. Stat..

[3] Akaike H., Parzen E., Tanabe K., Kitagawa G. (1998). Information theory and an extension of the maximum likelihood principle. Selected Papers of Hirotugu Akaike.

[4] Akaike H. (1974). A new look at the statistical model identification. IEEE Trans. Autom. Control.

[5] Hodges J.S., Sargent D.J. (1988). Counting degrees of freedom in hierarchical and other richly parameterised models. Biometrika.

[6] Schwarz G. (1978). Estimating the dimension of a model. Ann. Stat..

[7] Pan W. (1999). Bootstrapping likelihood for model selection with small samples. J. Comput. Gr. Stat..

[8] Rissanen J. (1987). Stochastic complexity. J. R. Stat. Soc..

[9] Rissanen J. (1996). Fisher information and stochastic complexity. IEEE Trans. Inf. Theory.

[10] Grünwald P., Grünwald P., Myung I., Pitt M. (2005). A tutorial introduction to the minimum description length principle. Advances in Minimum Description Length: Theory and Applications.

[11] Balasubramanian V. (1997). Statistical inference, Occam’s razor, and statistical mechanics on the space of probability distributions. Neural Comput..

[12] Kent J.T. (1982). The Fisher–Bingham distribution on the sphere. J. R. Stat. Soc..

[13] Boothby W.M. (2002). An Introduction to Differentiable Manifolds and Riemannian Geometry.

[14] Barron A., Rissanen J., Yu B. (1998). The minimum description length principle in coding and modeling. IEEE Trans. Inf. Theory.

[15] Rissanen J. (2000). MDL denoising. IEEE Trans. Inf. Theory.

[16] Wallace C.S., Dowe D.L. (1999). Refinements of MDL and MML coding. Comput. J..

[17] Wallace C.S., Boulton D.M. (1968). An information measure for classification. Comput. J..

[18] Wallace C.S. (2005). Statistical and Inductive Inference by Minimum Message Length.

[19] Lebanon G. (2006). Metric learning for text documents. IEEE Trans. Pattern Anal. Mach. Intell..

[20] Srivastava A., Jermyn I., Joshi S. (2007). Riemannian analysis of probability density functions with applications in vision. Proceedings of the IEEE Conference on Computer Vision and Pattern Recognition.

[21] Peter A., Rangarajan A. (2009). Information geometry for landmark shape analysis: Unifying shape representation and deformation. IEEE Trans. Pattern Anal. Mach. Intell..

[22] Peter A., Rangarajan A. (2008). Maximum likelihood wavelet density estimation with applications to image and shape matching. IEEE Trans. Image Process..

[23] Bingham C. (1964). Distributions on the Sphere and on the Projective Plane. Ph.D. Thesis.

[24] Parthasarathy B., Kadane J.B. (1991). Laplace approximations to posterior moments and marginal distributions on circles, spheres, and cylinders. Can. J. Stat..

[25] Kume A. (2005). Saddlepoint approximations for the Bingham and Fisher-Bingham normalising constants. Biometrika.

[26] Hall P., Hannan E. (1988). On stochastic complexity and nonparametric density estimation. Biometrika.

[27] Kontkanen P. (2009). Computationally Efficient Methods for MDL-Optimal Density Dstimation and Data Clustering. Ph.D. Thesis.

[28] Davies L., Gather U., Nordman D., Weinert H. (2009). A comparison of automatic histogram constructions. ESAIM.

[29] McKay D. (1992). A practical Bayesian framework for backpropation networks. Neural Comput..

[30] Stigler S.M. (1986). The History of Statistics: The Measurement of Uncertainty before 1900.

[31] Rao C.R. (1945). Information and accuracy attainable in estimation of statistical parameters. Bull. Calcutta Math. Soc..

[32] Jeffreys H. (1961). Theory of Probability.

[33] Kass R.E. (1989). The geometry of asymptotic inference. Stat. Sci..

[34] Barndorff-Nielsen O., Cox D., Reid N. (1986). The role of differential geometry in statistical theory. Int. Stat. Rev..

[35] Amari S.I., Nagaoka H. (2001). Methods of Information Geometry.

[36] Cover T., Thomas J. (2006). Elements of Information Theory.

[37] Laplace P. (1986). Memoir on the probability of the causes of events. Stat. Sci..

[38] Bertsekas D.P. (1999). Nonlinear Programming.

[39] Berry A.C. (1941). The accuracy of the Gaussian approximation to the sum of independent variates. Trans. Am. Math. Soc..

[40] Robert C., Casella G. (2004). Monte Carlo Statistical Methods.

[41] Heck D.W., Moshagen M., Erdfelder E. (2014). Model selection by minimum description length: Lower-bound sample sizes for the Fisher information approximation. J. Math. Psychol..

[42] Navarro D.J. (2004). A note on the applied use of MDL approximations. Neural Comput..

[43] Peter A., Rangarajan A. (2011). An information geometry approach to shape density minimum description length model selection. Proceedings of the IEEE International Conference on Computer Vision (ICCV) Workshops.

[44] Taylor C. (1987). Akaike’s information criterion and the histogram. Biometrika.

[45] Abramowitz M., Stegun I.A. (1965). Handbook of Mathematical Functions: With Formulas, Graphs, and Mathematical Tables.

[46] Marron S.J., Wand M.P. (1992). Exact mean integrated squared error. Ann. Stat..

[47] Wand M.P., Jones M.C. (1993). Comparison of smoothing parameterizations in bivariate kernel density estimation. J. Am. Stat. Assoc..

